# Awareness and Underlying Factors for Total Knee Arthroplasty Reluctance in Saudi Arabia

**DOI:** 10.7759/cureus.67942

**Published:** 2024-08-27

**Authors:** Talal A Alkindy, Amirah Abdullah A Alatawi, Meshari Salman A Alhawiti, Osama Nasser A Alayda, Randa Abdullah E Altuwaijri, Abdulrahman Abdullah S Alelyani, Abdullah Nasser A Alayda, Abdulrahman Mushabab A Almurayeh

**Affiliations:** 1 Orthopedics, Faculty of Medicine, University of Tabuk, Tabuk, SAU; 2 Medicine and Surgery, Faculty of Medicine, University of Tabuk, Tabuk, SAU; 3 Medicine and Surgery, Faculty of Medicine, Qassim University, Qassim, SAU; 4 Medicine and Surgery, Faculty of Medicine, King Khalid University, Abha, SAU

**Keywords:** total knee arthroplasty, reluctance, knowledge, expectation, awareness

## Abstract

Background

Total knee arthroplasty (TKA) is a successful surgical intervention for advanced knee arthritis. The efficacy of TKA in reducing pain and restoring joint function has been well documented. Despite the rewarding outcomes of TKA for knee osteoarthritis patients, their willingness to consider the procedure is limited.

Aim

This study aimed to assess patients' awareness and knowledge of total knee arthroplasty benefits and complications. Further, the reasons and factors contributing to reluctance among orthopedic patients in Saudi Arabia should be determined.

Methods

An online, structured, and self-administered questionnaire was used to collect data from adult orthopedic patients of both genders who were reluctant to undergo total knee arthroplasty despite surgeons’ recommendations. The online questionnaire link was shared across multiple platforms, orthopedic forums, and healthcare social media channels. Qualitative data were presented as frequencies and percentages, while continuous data were reported as the mean (standard deviation [SD]). The statistical package for the social sciences software program was used for statistical analysis.

Results

A total of 629 participants were involved. The awareness of the expected benefits score, on a scale from 7 to 35, showed a mean (SD) of 20.9 (5.6). The score of the attitude towards expected complications, on a scale from 5 to 25, had a mean (SD) of 15.2 (3.6). The attitude towards the expected complications showed a significantly higher mean (SD) score in the older group aged >60 years than the younger one aged <40 years (15.7 (4.1) vs. 14.9 (3.5), respectively). Likewise, overweight and obese participants showed a significantly higher mean (SD) expected complications score compared to the healthy and underweight ones (15.4 (3.7) vs. 14.8 (3.5), respectively). The recorded reasons for refusal to undergo TKA were fear of anesthesia complications (317, 50.4%), followed by financial limitations (245, 39.0%), the unavailability of experienced surgeons (232, 36.9%), and fear of unfavorable outcomes (189, 30.0%).

Conclusion

There was a gap in knowledge and awareness of total knee arthroplasty among orthopedic patients in Saudi Arabia. Perceptions of benefits were inadequate, and there were misconceptions about the expected complications. The level of expected complications was higher among elderly and obese patients. Furthermore, fear of anesthesia complications and unfavorable outcomes, in addition to economic and financial problems, constituted major barriers to undergoing the procedure.

## Introduction

Total knee arthroplasty (TKA) is one of the most commonly performed orthopedic procedures, and it involves surgical removal of the diseased articular surfaces of the knee, followed by resurfacing with prosthetic components [1]. A pivotal role of this procedure is to alleviate pain and enhance functionality. The increased mobility and independence help the patients engage in various activities and thereby improve their quality of life [2].

Patients with a variety of orthopedic conditions might need surgical intervention by TKA; however, osteoarthritis is the most common. Knee joint osteoarthritis is a very common orthopedic condition in Saudi Arabia, with a prevalence rate between 13% and 30%, and it places a high burden on the patients and health care system [3].

Total knee arthroplasty is an elective procedure that is reserved for patients with end-stage degenerative knee osteoarthritis. It is often indicated for those suffering from persistent chronic debilitating symptoms despite pharmacological, physical, occupational, and weight management treatment modalities [4].

Despite the rewarding outcomes of TKA for knee osteoarthritis patients, Bendich et al. [5] reported a restricted willingness to consider arthroplasty in a cohort of individuals diagnosed with knee osteoarthritis. They also highlighted the importance of recognizing and counseling the factors that influence a patient's decision to undergo TKA surgery. Li et al. [6] also detected reluctance to undergo TKA, reporting that only 33.8% of osteoarthritis patients were ready for surgery. Another study in Kuwait reported that fear of the procedure was the main reason for a longer delay in undergoing TKS despite clinicians' recommendations [7]. A study in China also concluded that despite the recognition of the advantages of total joint replacement surgery in treating arthritis, patients expressed worries about being crippled or suffering pain after the operation [8].

Patients’ knowledge is very crucial to optimizing TKA decision-making and outcomes. The existing gap between patients' and physicians' perceptions of the need, benefits as well as risks of surgery explains the observed reluctance to undergo knee arthroplasty. Understanding patients' decision-making processes and the reasons for delaying joint replacement surgery is essential. Educational interventions addressing socioeconomic considerations and the psychological readiness of the patient are of paramount importance [9-11].

Analyzing knee arthroplasty reluctance in Saudi Arabia is pivotal due to the rising incidence of joint replacement procedures [12]. Therefore, the present study aimed to assess patients' awareness and knowledge of total knee arthroplasty benefits and complications. Further, the reasons and factors contributing to reluctance among orthopedic patients in Saudi Arabia should be determined.

## Materials and methods

Study design, settings, and date

This cross-sectional study covered diverse regions in Saudi Arabia. It was conducted between April and June 2024.

Ethical considerations

This study was carried out after obtaining ethical approval from the Research Ethics Committee of the University of Tabuk, Saudi Arabia. We informed the participants that their participation was voluntary, they had the right to withdraw anytime without completing the questionnaire, and that their responses would be anonymous and confidential. Informed consent was signed before collecting data.

Sample size and sampling technique

The sample size was calculated using web-based Raosoft Software, Inc., Seattle, WA, USA (Raosoft, http://www.raosoft.com/samplesize.html). According to a 5% margin of error, a 95% confidence interval, and an anticipated response rate of 50% (as the actual prevalence is unknown), the minimum required sample size is 377 participants. A convenience sampling technique was applied to enroll the participants.

Inclusion criteria

We included adult orthopedic patients of both genders who were advised to undergo total knee arthroplasty but were reluctant to do so. Both the citizen and resident populations in Saudi Arabia were included.

Exclusion criteria

Patients with a history of orthopedic surgery and total, as well as subtotal knee replacement, were excluded.

Research instrument

The questionnaire was based on validated questionnaires used in relevant previously published research articles [13-15]. Two arthroplasty consultants reviewed the questionnaire to ensure question clarity and validity. It consists of two parts. The first part focused on the sociodemographic characteristics of the participants, while the second part collected information on four domains: current difficulties, expected benefits after surgery, expected complications, and general health. Each question in every domain was rated on a five-point Likert scale, ranging from 'strongly agree' to 'strongly disagree.' The domain of current difficulties included questions about pain and disability while walking, difficulties with stairs, the presence of knee pain, interference with daily activities, and self-care difficulties. The highest possible score for this domain is 30 points, while the lowest possible score is 6 points. The domain of expected benefits comprised inquiries regarding various physical activities such as sitting cross-legged, running, jumping, kneeling, lying, going up and down stairs, and walking long distances after the procedure. Additionally, it included perceptions about the quality of life after the arthroplasty. The highest possible score for this domain is 35 points, while the lowest possible score is 7 points. The domain of expected complications included concerns about persistent pain after the procedure, postoperative pain, an inability to walk, prolonged bed rest, and dangerous complications. The highest possible score for this domain is 25 points, while the lowest is 5 points. The general health domain comprised two questions that evaluated the patient's psychological status and overall health. The highest possible score is 10 points, while the lowest is 2 points. Additionally, the questionnaire included an inquiry about the suitability of surgical intervention if non-surgical options prove ineffective. It also featured a multiple-choice question that aimed to explore the reasons underlying a patient's refusal to undergo total knee arthroplasty. The researchers translated the questionnaire into Arabic and revised it, and it was pre-tested on a small sample to refine any problematic items.

Data collection

A reliable survey platform was used to design an online, structured, and self-administered questionnaire. The online questionnaire link was shared across multiple platforms, orthopedic forums, and healthcare social media channels.

Statistical analysis

All data were tabulated and analyzed by the statistical package for the social sciences software program, IBM SPSS Statistics for Windows, version 27 (IBM Corp., Armonk, NY, USA). The total score for each domain was calculated by giving 5 points for “strongly agree,” 4 points for “agree,” 3 points for “neutral,” 2 points for “disagree,” and 1 point for “strongly disagree.” Reverse scoring was required for items 3 and 5 in the current difficulties domain and item 1 in the general health domain. We presented qualitative data in terms of frequencies and percentages. Continuous variables were tested for distribution using the Shapiro-Wilk test and reported as mean ± standard deviation. We used age groups, gender, residence, marital status, education, obesity, and the question “Do you think surgical intervention is the best treatment if non-surgical options don’t work?” to investigate the factors related to awareness of the expected benefits and complications of total knee arthroplasty. This was accomplished by independent T and one-way analysis of variance (ANOVA) tests. Additionally, Pearson correlations between the total health score and expected benefits and complications scores were applied. A p-value < 0.05 was considered statistically significant.

## Results

A total of 629 participants were involved in the present study. About 411 (65.3%) were females, while 218 (34.7%) were males. There was a comparable distribution of all age groups except the “> 60” age group, which included only 42 (6.7%) participants. Most of them (609, 96.8%) were Saudi, and 340 (54.1%) were married. A total of 600 (95.4%) members were residing in the city, and 29 (4.6%) were in rural residences. More than half (59.5%) had studied up to the university level, 170 (27.0%) had a high school educational level, and 39 (6.2%) were postgraduates. Further, overweight and obese subjects constituted 34.6% (212/612) and 26.3% (161/612), respectively (Table 1).

**Table 1:** Sociodemographic characteristics of the study participants. N: number; %: percentage; BMI: body mass index; a denotes missing data as a few participants did not provide information about their weight and/or height.

Variables		N = 629	%
Age (years)	18–20	121	19.2%
	>20 to 30	146	23.2%
	>30 to 40	106	16.9%
	>40 to 50	132	21.0%
	>50 to 60	82	13.0%
	>60	42	6.7%
Gender	Female	411	65.3%
	Male	218	34.7%
Nationality	Saudi	609	96.8%
	Non-Saudi	20	3.2%
Residence	City	600	95.4%
	Rural	29	4.6%
Marital status	Married	340	54.1%
	Non-married	289	45.9%
Education	Less than high school	46	7.3%
	High school	170	27.0%
	Bachelor's degree	374	59.5%
	Postgraduate studies	39	6.2%
BMI, kg/m^2 ^		N = 612^a^	%
	Underweight (<18.5)	47	7.7%
	Healthy (18.5–24.9)	192	31.4%
	Overweight (25.0–29.9)	212	34.6%
	Obese (≥30.0)	161	26.3%

Inquiries about the current difficulties experienced by the studied patients revealed feeling pain in the knee (303, 48.2%), pain during walking (251, 39.9%), difficulties in going up and downstairs (268, 42.6%), and only 84 (13.4%) felt difficulties in taking care of themselves. Further, 263 (41.8%) reported that the knee joint does not disable them while walking, and 278 (44.2%) conveyed that knee pain does not interfere with daily activities. The total score of the current difficulties ranged from 6.0 to 30.0, with a mean of 17.2 ± 4.7 (Table 2).

**Table 2:** Current difficulties experienced by the studied patients. N: number; %: percentage; SD: standard deviation.

Variables		N = 629	%
I feel pain in my knee	Strongly agree	103	16.4%
	Agree	200	31.8%
	Neutral	159	25.3%
	Disagree	97	15.4%
	Strongly disagree	70	11.1%
I feel pain when walking	Strongly agree	78	12.4%
	Agree	173	27.5%
	Neutral	171	27.2%
	Disagree	135	21.5%
	Strongly disagree	72	11.4%
My knee does not disable me while walking	Strongly agree	115	18.3%
	Agree	148	23.5%
	Neutral	153	24.3%
	Disagree	140	22.3%
	Strongly disagree	73	11.6%
I have difficulties in going up and downstairs	Strongly agree	100	15.9%
	Agree	168	26.7%
	Neutral	141	22.4%
	Disagree	116	18.4%
	Strongly disagree	104	16.5%
Knee pain does not interfere with my daily activities	Strongly agree	109	17.3%
	Agree	169	26.9%
	Neutral	145	23.1%
	Disagree	144	22.9%
	Strongly disagree	62	9.9%
I have difficulties in taking care of myself	Strongly agree	28	4.5%
	Agree	56	8.9%
	Neutral	111	17.6%
	Disagree	210	33.4%
	Strongly disagree	224	35.6%
Total score	Minimum–maximum	6.0–30.0	
	Mean ± SD	17.2 ± 4.7	

A total of 158 (25.1%) participants recognized that they could sit cross-legged after the procedure, and 149 (23.7%) participants agreed that the procedure helped them to run and jump. Some participants reported other benefits of the procedure, including the ability to kneel and lie (195, 31%), flex the knee (197, 31.3%), go up and down stairs (202, 32.1%), and walk long distances (196, 31.2%). Furthermore, 407 (64.7%) participants documented that total knee arthroplasty would improve the quality of their lives. The awareness of the expected benefits score showed a mean of 20.9 ± 5.6 (Table 3).

**Table 3:** Awareness of the expected benefits after knee arthroplasty procedure. N: number; %: percentage; SD: standard deviation.

Variables		N = 629	%
Can sit cross-legged after the procedure?	Strongly agree	42	6.7%
	Agree	116	18.4%
	Neutral	173	27.5%
	Disagree	212	33.7%
	Strongly disagree	86	13.7%
Can run and jump after the procedure?	Strongly agree	35	5.6%
	Agree	114	18.1%
	Neutral	155	24.6%
	Disagree	242	38.5%
	Strongly disagree	83	13.2%
Can kneel and lie after the procedure?	Strongly agree	70	11.1%
	Agree	125	19.9%
	Neutral	176	28.0%
	Disagree	203	32.3%
	Strongly disagree	55	8.7%
Can flex the knee after the procedure?	Strongly agree	56	8.9%
	Agree	141	22.4%
	Neutral	202	32.1%
	Disagree	179	28.5%
	Strongly disagree	51	8.1%
Can go up and down stairs after the procedure?	Strongly agree	52	8.3%
	Agree	155	24.6%
	Neutral	225	35.8%
	Disagree	160	25.4%
	Strongly disagree	37	5.9%
Can walk long distances after the procedure?	Strongly agree	51	8.1%
	Agree	145	23.1%
	Neutral	182	28.9%
	Disagree	206	32.8%
	Strongly disagree	45	7.2%
Total knee arthroplasty will improve the quality of my life	Strongly agree	170	27.0%
	Agree	237	37.7%
	Neutral	144	22.9%
	Disagree	60	9.5%
	Strongly disagree	18	2.9%
Total score	Minimum–maximum	7.0–35.0	
	Mean ± SD	20.9 ± 5.6	

Regarding the perceptions of the studied patients about the expected complications after a knee arthroplasty procedure, some participants thought that pain would persist after the procedure (179, 28.4%), they would feel severe postoperative pain (337, 53.5%), they would not be able to move after surgery (189, 30%), or they would be bedridden for a long time after surgery (117, 18.6%). Furthermore, 221 (35.2%) reported that the procedure had dangerous complications such as bleeding, nerve injury, infections, etc. The mean score of the attitude toward expected complications was 15.2 ± 3.6 (Table 4).

**Table 4:** Perceptions of the studied patients about the expected complications after knee arthroplasty procedure. N: number; %: percentage; SD: standard deviation.

Variables		N = 629	%
Pain will persist after the procedure	Strongly agree	36	5.7%
	Agree	143	22.7%
	Neutral	248	39.4%
	Disagree	176	28.0%
	Strongly disagree	26	4.1%
I will feel a severe postoperative pain	Strongly agree	150	23.8%
	Agree	187	29.7%
	Neutral	223	35.5%
	Disagree	52	8.3%
	Strongly disagree	17	2.7%
I will not be able to move after surgery	Strongly agree	46	7.3%
	Agree	143	22.7%
	Neutral	209	33.2%
	Disagree	182	28.9%
	Strongly disagree	49	7.8%
I will be bedridden long time after surgery	Strongly agree	29	4.6%
	Agree	88	14.0%
	Neutral	166	26.4%
	Disagree	280	44.5%
	Strongly disagree	66	10.5%
The procedure has dangerous complications such as bleeding, nerve injury, infections, etc.	Strongly agree	57	9.1%
	Agree	164	26.1%
	Neutral	205	32.6%
	Disagree	169	26.9%
	Strongly disagree	34	5.4%
Total score	Minimum–maximum	5.0 - 25.0	
	Mean ± SD	15.2 ± 3.6	

The expected benefits awareness score was comparable in the age groups <40 and >60 (21.5 ± 5.8 vs. 21.5 ± 5.5, respectively), while it was significantly lower in the middle age group between 40 and 60 (P < 0.05). Further, participants who thought that surgical intervention was the best treatment if non-surgical options did not work showed significantly higher awareness of the expected benefits than their counterparts (21.2 ± 5.5 vs. 19.4 ± 6.1, respectively). Moreover, the attitude toward the expected complications showed a significantly higher mean score in the older group aged > 60 years (15.7 ± 4.1) than in the younger one aged <40 years (14.9 ± 3.5). Females also showed a significantly higher mean expected complications score than males (15.5 ± 3.5 vs. 14.6 ± 3.7, respectively). Likewise, overweight and obese participants showed a significantly higher mean expected complications score compared to the healthy and underweight ones (15.4 ± 3.7 vs. 14.8 ± 3.5, respectively) (Table 5).

**Table 5:** Factors associated with awareness of the benefits and complications of knee arthroplasty. SD: standard deviation ^a^Variables that showed significant differences in benefits awareness score ^b^Variables that showed significant differences in complications awareness score, the cutoff of significance at p < 0.05.

Variables		Benefits awareness score		Complications awareness score	
		Mean	SD	Mean	SD
Age, years^ a,b^	≤40	21.5	5.8	14.9	3.5
	>40 to 60	19.9	5.1	15.6	3.6
	>60	21.5	5.5	15.7	4.1
Gender^b^	Female	21.1	5.5	15.5	3.5
	Male	20.6	5.8	14.6	3.7
Residence	City	21.0	5.6	15.2	3.6
	Rural	19.7	6.3	15.4	4.0
Marital status	Married	20.8	5.5	15.1	3.6
	Non-married	21.2	5.8	15.3	3.6
Education	Below university	21.0	5.5	15.5	3.7
	University and postgraduate	20.9	5.7	15.0	3.5
Obesity^b^	Healthy and underweight	21.3	5.8	14.8	3.5
	Overweight and obese	20.7	5.5	15.4	3.7
Do you think surgical intervention is the best treatment if non-surgical options don’t work?^a^	No	19.4	6.1	15.5	4.3
	Yes	21.2	5.5	15.1	3.5

**Figure 1:**
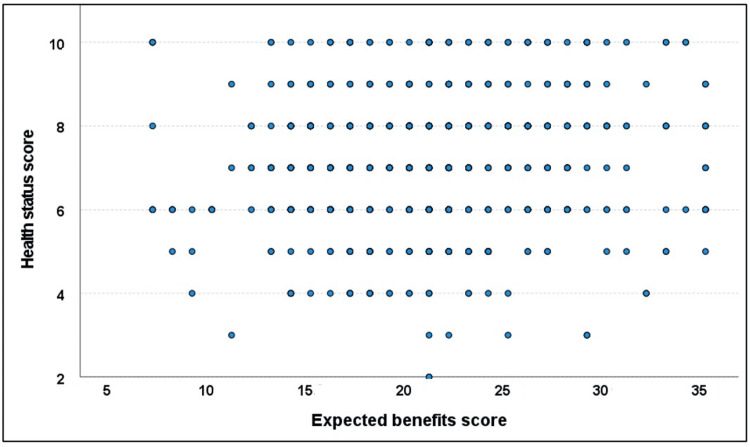
Correlation between health status and expected benefits scores.

**Figure 2:**
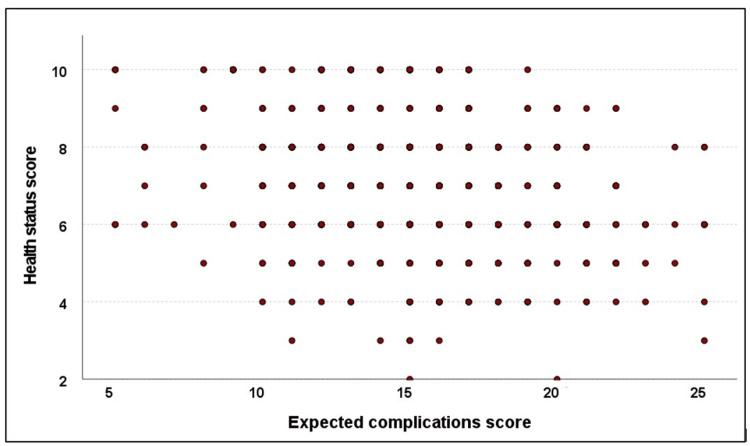
Correlation between health status and expected complications scores.

The total health score ranged from 2 to 10, with a mean of 7.0 ± 1.6. It showed a significant weak positive correlation with the expected benefits score (r = 0.096, p = 0.016) but a significant weak negative correlation with the expected complications score (r = −0.263, p < 0.001) (Figures 1-2).

**Figure 3:**
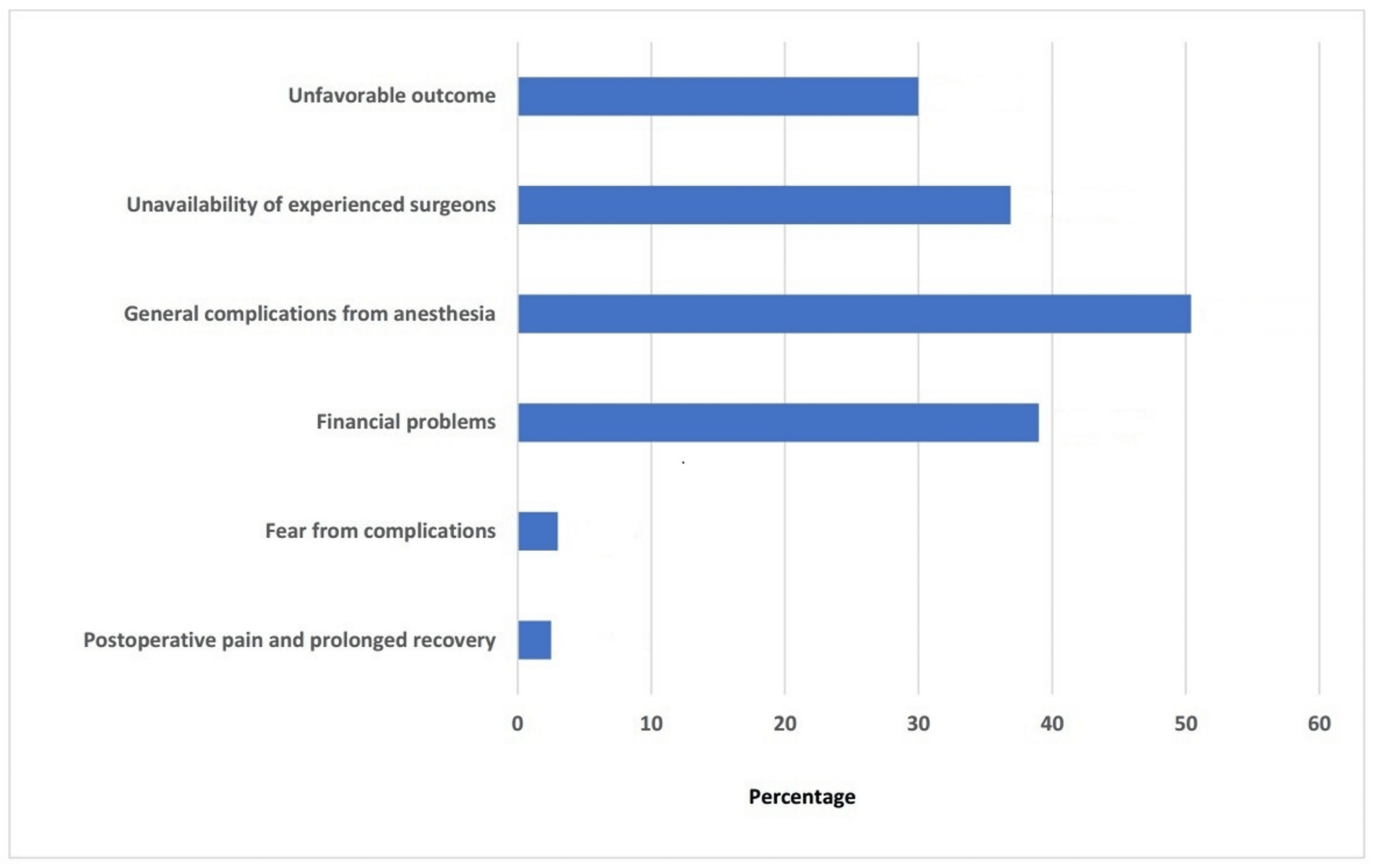
Reasons for refusal to undergo total knee arthroplasty.

Regarding the reasons for refusal to undergo total knee arthroplasty, general complications from anesthesia were the most frequent (317, 50.4%), followed by the financial limitations to undergo the surgery (245, 39%). The unavailability of experienced surgeons was mentioned by 36.9% (232/629), and fear of unfavorable outcomes represented 30% (189/629). Other explanations were fear of complications, postoperative pain, and prolonged recovery, which were noted in a few participants: 3% (19/629) and 2.5% (16/629), respectively (Figure 3).

## Discussion

Total knee arthroplasty is a medical advancement that quickly gained widespread acceptance and is now commonly acknowledged as an effective intervention for advanced knee arthritis. The efficacy of TKA in reducing pain and restoring joint function has been well documented [16]. Willingness to consider surgery is the most protuberant indicator for referral to TKA [17].

Awareness enables patients to make informed choices about their healthcare. Kwoh et al. [18] emphasized the relationship between knowledge of TKA and the willingness to undergo surgery. Furthermore, patients’ expectations regarding the surgical procedure have a great influence on their decisions, outcomes, satisfaction, and quality of life [18].

One of the aims of this study was to explore the knowledge and attitude of orthopedic patients towards total knee arthroplasty in Saudi Arabia. The present study revealed an inadequate level of knowledge about the expected benefits of TKA. The mean knowledge score, on a scale from 7 to 35 points, was 20.9 ± 5.6. This finding agrees with previous studies in Saudi Arabia, which revealed a considerable lack of knowledge among the public about TKA [19,20]. Likewise, a study in Hail, Saudi Arabia, detected an average awareness score of 15 out of a total of 34 points, which is lower than ours [15].

The studied patients' expectations of benefits were low. Only one-fourth (158, 25.1%) recognized that they could sit cross-legged after the procedure, and 23.7% (149/629) agreed that the procedure helped them to run and jump. There was also a lack of knowledge of other benefits, such as the ability to kneel and lie (195, 31%), flex the knee (197, 31.3%), go up and down stairs (202, 32.1%), and walk long distances (196, 31.2%). In this context, Al-Mohrej et al. [19] investigated the expectations of the general population after undergoing total joint replacement. About 9% (138/1540) thought that pain would resolve completely within a short duration after the surgery, and around 22% (338/1540) thought they could pray normally after the surgery. Alternatively, Almaawi et al. [21] reported higher expectations of the population in terms of pain relief and improvement in the quality of life (329/486, 67.7% and 399/486, 82%, respectively).

According to the current findings, the level of awareness about TKA complications was also inadequate. The mean score was 15.2 ± 3.6 on a scale from 5 to 25. The perceptions of the studied patients about the expected complications after the knee arthroplasty procedure were severe postoperative pain (337, 53.5%), inability to move after surgery (189, 30%), and surgery-related bleeding, nerve injury, and infections (221, 35.2%). Similarly, Almaawi et al. [21] reported an average level of awareness about the complications of total joint replacement, with surgical site infection (282/486, 58%) and knee instability (281/486, 57.8%) being the most frequently reported.

The present study also showed that the level of the expected complication significantly increased in elderly people aged more than 60 years, females, as well as obese and overweight members. This is in line with Abd-Allah et al. [13], who reported unsatisfactory knowledge and increased concerns about total joint replacement among elderly patients. Their concerns included intolerance of surgery, inability to move after the procedure, persistent postoperative pain, and fear of being bedridden. Alternatively, Alshammari et al. [15] reported that education is the only significant predictor of awareness. Furthermore, it has been reported that obese and overweight patients ultimately experience more pain and greater difficulties, which might affect their expectations of surgery [14].

The physical and psychological health of the studied patients showed a weak impact on the expected benefits and complications of TKA. Good health status was associated with high expectations of benefits and low expectations of complications. Healthcare providers should take into account the general health and psychological aspects of the candidate patients during preoperative counseling. This is supported by Larsson et al. [22], who concluded that the patient's participation in their care increases their sense of safety and promotes postoperative recovery [22].

Inquiries about reasons for reluctance to undergo TKA explored that fear of anesthesia complications is a major concern (317, 50.4%). Economic and financial problems (245, 39%), the unavailability of experienced surgeons (232, 36.9%), and fears of unfavorable outcomes (189, 30%) also affect the decision of the operation. Other barriers included fear of postoperative complications and pain. Correspondingly, Almaawi et al. [21] explored the misconception that surgery is not beneficial (288/472, 61%), persistent pain (164/486, 33.7%), complications from general anesthesia (123/473, 26%), and inability to attain an experienced surgeon (116/478, 24.1%) as the factors that preclude patients from undergoing total joint replacement. Furthermore, Ahmed et al. [23] reported a variety of social (16/65, 24.61%), economic (25/65, 38.46%), and religious (10/65, 15.38%) reasons in addition to fear of complications (14/64, 21.88%). Their participants also gave several explanations, frequently combining worries about potential problems with financial worries (37/65, 56.9%). The observed worries of the patients emphasize the need for all-encompassing patient counseling and education. McDonald et al. [24] emphasized the importance of discussing all details with the patient, including the surgical procedure steps, postoperative care, potential surgical and nonsurgical complications, postoperative pain management, movements to avoid after surgery, and favorable outcomes.

The study has several limitations, including its reliance on an online survey and self-reporting of data, which potentially introduces selection and recall bias that impacts data accuracy. However, it enabled us to cover different geographical regions in Saudi Arabia that represent the diversity of knowledge and attitudes toward total knee arthroplasty in the entire country. In addition, the study's cross-sectional design impedes instituting causal relationships between knowledge and various socioeconomic and health status factors.

## Conclusions

The present findings indicate a substantial gap in knowledge and awareness of total knee arthroplasty among orthopedic patients in Saudi Arabia. Perceptions of benefits were inadequate, and there were misconceptions about the expected complications. The level of expected complications was higher among the elderly, females, and obese patients. Furthermore, fear of anesthesia complications and unfavorable outcomes, in addition to the economic and financial problems, constituted major barriers to undergoing total knee arthroplasty. Preoperatively targeted patient education to provide accurate information and discuss any misconceptions about total knee arthroplasty is vital for promoting informed healthcare decision-making. This ultimately improves healthcare outcomes and reduces the burden on healthcare systems.
